# Coordination of the maize transcriptome by a conserved circadian clock

**DOI:** 10.1186/1471-2229-10-126

**Published:** 2010-06-24

**Authors:** Sadaf Khan, Scott C Rowe, Frank G Harmon

**Affiliations:** 1 Department of Plant & Microbial Biology, University of California, Berkeley, CA, 94720, USA; 2 Plant Gene Expression Center, USDA-ARS, Albany, CA 94710, USA

## Abstract

**Background:**

The plant circadian clock orchestrates 24-hour rhythms in internal physiological processes to coordinate these activities with daily and seasonal changes in the environment. The circadian clock has a profound impact on many aspects of plant growth and development, including biomass accumulation and flowering time. Despite recent advances in understanding the circadian system of the model plant *Arabidopsis thaliana*, the contribution of the circadian oscillator to important agronomic traits in *Zea mays *and other cereals remains poorly defined. To address this deficit, this study investigated the transcriptional landscape of the maize circadian system.

**Results:**

Since transcriptional regulation is a fundamental aspect of circadian systems, genes exhibiting circadian expression were identified in the sequenced maize inbred B73. Of the over 13,000 transcripts examined, approximately 10 percent displayed circadian expression patterns. The majority of cycling genes had peak expression at subjective dawn and dusk, similar to other plant circadian systems. The maize circadian clock organized co-regulation of genes participating in fundamental physiological processes, including photosynthesis, carbohydrate metabolism, cell wall biogenesis, and phytohormone biosynthesis pathways.

**Conclusions:**

Circadian regulation of the maize genome was widespread and key genes in several major metabolic pathways had circadian expression waveforms. The maize circadian clock coordinated transcription to be coincident with oncoming day or night, which was consistent with the circadian oscillator acting to prepare the plant for these major recurring environmental changes. These findings highlighted the multiple processes in maize plants under circadian regulation and, as a result, provided insight into the important contribution this regulatory system makes to agronomic traits in maize and potentially other C4 plant species.

## Background

Plants match their physiology to daily and seasonal environmental changes through the circadian clock, an internal timekeeping mechanism that regulates a wide range of plant behavior. Overt circadian rhythms in plants include rhythmic leaf movements, stomatal conductance, and growth [1]. Rhythms are maintained with a period of approximately 24 hours in the absence of environmental cues and over the normal range of ambient temperatures. The circadian system enables plants to anticipate and synchronize their physiology to the recurring environmental changes brought on by day and night. The consequence of proper clock and environment synchronization is optimized fitness [2,3]. Beyond the daily control of plant biology, circadian rhythms also allow plants to track seasonal change according to day length, or photoperiod [4,5]. The interplay of the circadian clock and photoperiod allows photoperiod sensitive plants to initiate floral development in accordance with the season [6,7]. Thus, the circadian clock is an endogenous timer that maintains normal plant biology on both daily and seasonal timescales.

The circadian clock is best described in the model plant *Arabidopsis thaliana*. Arabidopsis mutants with impaired clock function show reduced fitness arising from mismatch between internal rhythms and external environmental conditions [8,9]. In addition, many circadian clock mutants exhibit alterations in flowering time associated with defects in day length perception [10]. At the molecular level, Arabidopsis circadian physiology requires the products of so-called core clock genes, whose mutation widely disrupts circadian physiology [11]. The core clock proteins regulate expression of one another in a network of feedback loops [12]. The myb-like transcription factors CCA1 and LHY directly control *TOC1*, *PRR7*, and *PRR9 *expression [13,14]. In turn, these pseudoresponse regulators define the expression patterns of CCA1 and LHY. In addition, the TCP transcription factor CHE serves as a transcriptional regulator of *CCA1 *[15]. Additionally, the clock-specific photoreceptor and F-box protein ZTL controls TOC1 function through 26S proteasome-mediated protein degradation of TOC1 at night [16,17]. The core circadian oscillator also requires the activity of ELF3 and LUX in the evening [18,19]. This regulatory network generates phase-specific 24-hour oscillations in each core clock gene, with the state of the overall system reflecting time of day.

Orthologs of circadian clock components in plants outside of Arabidopsis have been best characterized in rice [20] and *Lemna *[21]. The rice genome encodes single orthologs of CCA1 and GI, but two potential orthologs of ZTL. In addition, rice possesses five unique PRR orthologs, including a clear ortholog of TOC1 [22]. Overexpression of these rice orthologs in Arabidopsis modifies circadian rhythms, which is consistent with the function of these proteins being conserved between rice and Arabidopsis [20]. In addition, rice *TOC1 *and *PRR7 *partially complement the corresponding Arabidopsis *toc1 *and *prr7 *mutants [23]. Circadian clock related genes have also been described in two species of the monocot *Lemna*, including *LHY*, *GI*, *ELF3*, *TOC1*, and the other *PRRs *[21]. Knockdown and overexpression of *LHY*, *ELF3*, and *GI *in *Lemna *suggest the topology of the *Lemna *circadian system is conserved with Arabidopsis and rice [24].

Genome-wide expression assays have revealed details behind circadian clock regulation of overt plant physiology. In Arabidopsis, the core oscillator genes are only a fraction of the genes showing cyclic expression in constant conditions. Whole genome transcriptional profiling demonstrates that the steady state transcripts of ~30% of Arabidopsis genes cycle every 24 hours in constant conditions [25,26]. Clock controlled phasing of these cyclic genes throughout a day activates or represses metabolic and signal transduction pathways, thereby yielding macroscopic circadian rhythms in plant physiology. Genes involved in related pathways share timing of peak expression; for example, genes encoding enzymes in secondary metabolite biosynthesis, nutrient assimilation, and hormone signaling are co-regulated by the circadian clock [27-29].

Approximately half of the world's grass species employ C4 photosynthesis, including the food crops maize, sugarcane, and sorghum, as well as the potential biofuel crops switchgrass and *Miscanthus*. C4 plants capture CO_2 _as the 4-carbon compound oxaloacetate in specialized mesophyll cells and the newly captured carbon is then transported into adjacent bundle sheath cells to enter the Calvin cycle through the action of Rubisco. Primary carbon fixation in C3 plants like Arabidopsis occurs in mesophyll cells through Rubisco-mediated incorporation of CO_2 _into the 3-carbon compound 3-phosphoglycerate. Direct CO_2 _capture by Rubisco reduces the photosynthetic efficiency of C3 plants because photosynthetic rate is limited both by CO_2 _diffusion from the atmosphere and by photorespiration that increases at low CO_2 _concentrations and high temperatures. The physical partitioning of CO_2 _capture and the Calvin cycle in C4 plants improves photosynthetic efficiency under low CO_2 _concentrations and reduces photorespiration by Rubisco at high temperatures and low CO_2 _concentrations. As a consequence, C4 crop plants assimilate biomass more efficiently than C3 plants at the high temperatures typical of agricultural settings [30].

Previous studies have shown the circadian clock serves to coordinate expression of genes encoding the photosynthesis apparatus in plants that carry out C3 photosynthesis [28]. The circadian system of C4 plants remains uncharacterized. Therefore, examination of the maize circadian system is fundamental to understanding the impact of circadian regulation on C4 photosynthesis, as well as the many other areas of maize metabolism where circadian rhythms are important. In this study, transcriptional profiling revealed the maize circadian transcriptome and this provided an initial characterization of the aspects of maize physiology under circadian clock influence.

## Results and Discussion

### Widespread circadian regulation of the maize transcriptome

To map the maize circadian transcriptome, mRNA levels in the aerial tissues of week-old maize B73 plants were determined by transcriptional profiling with the Affymetrix GeneChip^® ^Maize Genome Array. The B73 inbred is widely used and is the source material for the complete maize genome sequence [31]. Young plants were chosen as the experimental model because at this developmental stage all the tissues of the plant were easily sampled at once, unlike older maize plants that were too large to sample whole. Plants were exposed to 12 h light:12 h dark photocycles to set the circadian clock and then transferred to LL conditions for 48 additional hours. While in LL, aerial tissue was harvested every 4 hours and transcript levels in three pooled replicate samples were determined for the 13,339 maize genes represented on the microarray. The genes on this microarray account for approximately 41% of the entire maize genome, as the maize genome is predicted to have over 32,540 protein-encoding genes [31]. Consequently, the genes with circadian expression identified here may represent nearly half of the number of predicted maize genes with rhythmic expression.

**Figure 1:**
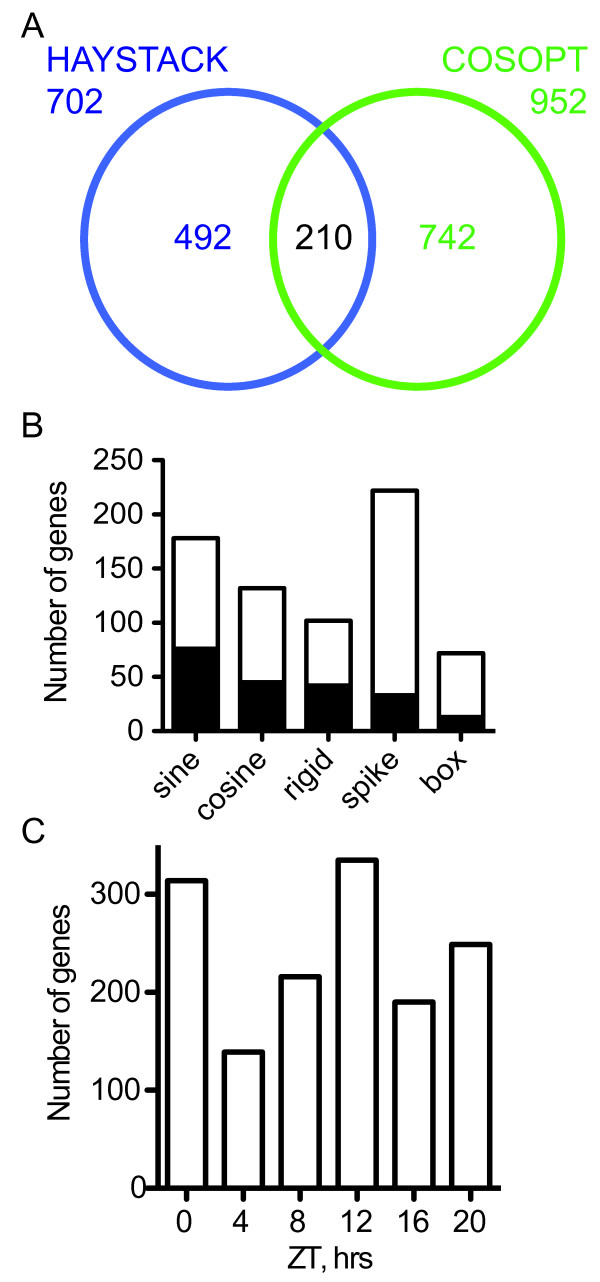
**A large fraction of maize genes exhibited circadian expression patterns and expression occurred at all phases**. A) Venn diagram of the overlap between transcripts found to have circadian waveforms by HAYSTACK (blue) and COSOPT (green). Union is shown in black. B) Waveforms of overlapping (black bars) and unique (white bars) cycling probe sets identified by HAYSTACK compared to those found by COSOPT. C) The majority of maize transcripts were preferentially expressed at morning (ZT0) or midnight (ZT12).

Following preprocessing and normalization of the raw hybridization values, genes on the array exhibiting circadian waveforms were identified by two well-established methods known as COSOPT and HAYSTACK [26,32]. COSOPT is a method that fits gene expression profiles to a series of modified cosine models, with the agreement between the experimental data and the model reported by the MMC-β value [32]. Lower values of MMC-β indicate a better fit to the cosine model. 952 genes were considered to exhibit a circadian waveform based on a MMC-β cutoff value of ≤ 0.05 (Figure 1A), which is a cutoff established by previously published reports for analogous circadian datasets in Arabidopsis and mouse [25,26,28,33,34]. The HAYSTACK tool identifies rhythmic waveforms by determining the agreement between expression profiles and a collection of models, which represent different types of potential circadian expression patterns [35]. The degree of correlation between model and experimental waveforms is determined by Pearson's correlation coefficient, where an exact match corresponds to PCC = 1.0. Maize transcripts with expression patterns matching a model with a PCC value of 0.7 or greater were considered to have a circadian rhythm. Based on this criterion, HAYSTACK analysis called 702 transcripts as exhibiting a circadian rhythm (Figure 1A). PCC values of 0.7 or greater consistently selected good matches between models and experimental waveforms, but this cutoff is more relaxed than the 0.8 value applied previously for a large collection of Arabidopsis time courses [26].

Comparing the genes called rhythmic by COSOPT and HAYSTACK revealed that the two methods had 210 genes in common (Figure 1A), a proportion similar to that described for Arabidopsis circadian expression datasets [26]. COSOPT identified almost twice as many unique rhythmic transcripts as found by HAYSTACK. This outcome was unexpected, since HAYSTACK has the potential to match a larger diversity of waveforms [35] and HAYSTACK has been shown to identify a larger proportion of rhythmically expressed transcripts in Arabidopsis datasets, most notably those with expression patterns in the spike class [26]. Not surprisingly, HAYSTACK appeared to favor maize genes showing the spike and box waveforms over other waveform classes, like cosine that COSOPT was designed to find (Figure 1B). Thus, under these standard cutoffs COSOPT was more sensitive than HAYSTACK in finding transcripts that match a cosine waveform. Furthermore, the cosine waveform appeared to be the dominant pattern of expression in maize seedlings under LL conditions.

Collectively, COSOPT and HAYSTACK indicated 1,444 transcripts, or ~10% of the expressed genes on the microarray showed rhythmic expression (Additional file 1 Table S1); therefore, a substantial part of the maize transcriptome is subject to regulation by the circadian clock. Genes in this collection included known maize circadian clock-regulated transcripts such as putative maize flowering time genes *gi1A *and *conz1 *[36], the well-established circadian clock-regulated gene *cat3 *[37], and several *lhcb *genes [38] (Additional file 2 Figure S1). Assuming a maize genome of 32,540 genes [31], the full circadian transcriptome of maize potentially represents a minimum of 3,254 genes. This degree of clock regulation is comparable to that observed in Arabidopsis with a partial genome array [28] and in other model systems like *Drosophila melanogaster*, *Neurospora crassa*, and *Mus musculus *[39].

### Preferential expression of maize circadian gene expression at dawn and dusk

A fundamental role of the clock is to anticipate day and night; as a result, the majority of circadian gene expression in Arabidopsis, rice, and poplar is timed, or phased, to precede or coincide with these recurring environmental shifts [26,28]. To determine whether the maize circadian transcriptome was similarly organized, the cycling maize transcripts were sorted into six phase bins based on expression waveform. The six phase bins were distributed in 4-hour intervals throughout the 24-hour subjective day: dawn (ZT0 hours), midday (ZT4), late day (ZT8), dusk (ZT12), midnight (ZT16), and early morning (ZT20). K-means clustering successfully placed all transcripts into one of these six phase bins (Additional file 3 Figure S2), with the exception of four outliers. As expected, rhythmically expressed transcripts were preferentially phased to the transitions into or out of subjective day (Figure 1C). This distribution was significantly different from that expected by chance, as assessed by χ^2 ^testing (χ^2 ^= 109.83, P(χ^2^) = 4.45 × 10^-22^, df = 5, α = .05). The single largest phase bin was ZT12 with 335 of cycling genes, followed by ZT0 encompassing 314 transcripts (Figure 1C). The early morning phase bin was also predominant, containing 249 genes with peak expression at ZT20. The remaining transcripts were distributed almost equally over the remaining phase bins. This analysis indicated the maize circadian oscillator parses gene expression in accordance with predictable environmental changes associated with day and night.

### Phase-specific distribution of key physiological processes

The genes on the array were annotated to identify the maize cellular processes under clock control. The annotation process involved matching the probe sets on the array to maize genes and then extracting the GO Slim classification for each transcript provided by the Maize Sequence Consortium (full method is described in "Methods" and outlined in Additional file 4 Figure S3). Of the cycling transcripts, 655 were assigned to GO Slim classifications for Cellular Component, Biological Process, and Molecular Function (Additional file 5 Table S2). The cycling dataset was evaluated for overrepresentation of GO Slim categories to determine whether the maize circadian clock preferentially regulated specific plant processes. Applying hypergeometric distribution analysis to the entire cycling dataset showed the Molecular Function GO Slim terms "catalytic activity" (GO: 0003824), "binding" (GO: 0005488), and "transporter activity" (GO: 0005215) were overrepresented in this group of transcripts (Table 1). Each of these GO Slim terms indicates transcripts encoding enzymes, receptors, and proteins involved in movement of macromolecules, small molecules, and ions are an important aspect of the maize circadian transcriptome. In the Biological Process classification, genes annotated with the GO Slim terms ""generation of precursor metabolites and energy" (GO: 0006091), "carbohydrate metabolic process" (GO: 0005975), and "photosynthesis" (GO: 0015979) were enriched in the circadian expressed gene set (Table 1). Together, these three GO Slim terms suggested the maize circadian clock makes an important contribution to fitness by regulating energy and carbohydrate metabolism. Similar GO Slim terms were also found to be overrepresented in the Arabidopsis circadian transcriptome [26], which demonstrates the involvement of the plant circadian clock in these processes is highly conserved.

**Table 1:** The maize circadian system preferentially regulated expression of genes encoding components of energy and metabolism pathways

GO Slim Category			
GO Slim term	Description	^1^e-score	Phase
**Molecular Function**			

GO: 0003824	catalytic activity	0.0000178	all
GO:0005488	binding	0.00595	all
GO: 0005215	transporter activity	0.0204	all
			
**Biological Process**			

GO: 0006091	generation of precursor metabolites and energy	0.000451	all
GO: 0005975	carbohydrate metabolic process	0.000421	all
GO: 0008152	metabolic process	0.00757	all
GO: 0015979	photosynthesis	0.0200	all
			
^2^**Biological Process within phase group**			

GO: 0015979	photosynthesis	0.000806	4
GO: 0006519	cellular amino acid and derivative metabolic process	0.0318	20
GO: 0009058	biosynthetic process	0.0156	20

^1^Overrepresentation of GO Slim terms was calculated using hypergeometric distribution followed by a modified Bonfferoni correction for multiple testing as implemented in GeneMerge [72], where the e-score is comparable to a P-value. Overrepresented GO terms were those with e-scores < 0.05.

^2^Each phase group was analyzed separately to identify GO terms overrepresented at a specific phase.

**Figure 2:**
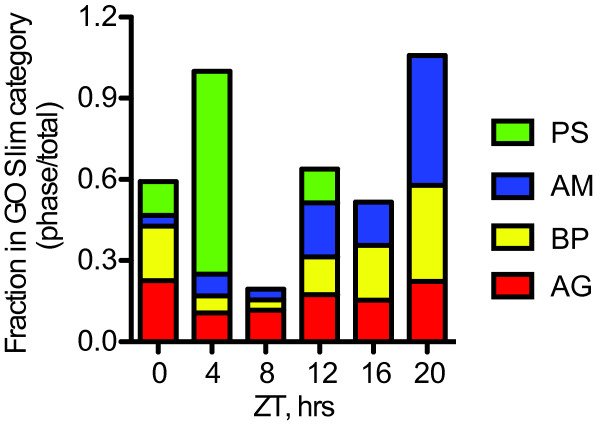
**The maize circadian system directed phase-specific expression of genes involved in biosynthesis**. Fraction of all genes in each phase cluster with GO Slim terms GO: 0015979 "photosynthesis" (PS, green), GO: 0006519 "cellular amino acid and derivative metabolic process" (AM, blue), and GO: 0009058 "biosynthetic process" (BP, yellow) compared to all genes on the microarray (AG, red). Hypergeometric distribution analysis from Table 2 indicated GO: 0015979 "photosynthesis" genes are overrepresented at midday (ZT4), whereas GO: 0006519 "cellular amino acid and derivative metabolic process" and GO: 0009058 "biosynthetic process" were enriched at early morning (ZT20).

Hypergeometric distribution analysis further tested whether expression of genes in particular GO Slim terms showed a time-of-day specific enrichment. The photosynthesis GO Slim term was enriched in the genes phased to ZT4 (Table 1). The bias toward photosynthesis genes at ZT4 is clear when the abundance of these genes is plotted as a function of phase (Figure 2). The Arabidopsis circadian clock similarly organizes photosynthesis-associated genes to reach peak expression early in the day [28]. Therefore, the circadian clock prepares maize plants for increasing illumination by up-regulating photosynthesis genes early in the light period. A phasing preference was also clear for the expression of genes associated with "cellular amino acid and derivative metabolic process" (GO: 0006519), which showed overrepresentation of genes with peak levels at ZT20 (Table 1, Figure 2). These results indicate maize protein synthesis may also have a pre-dawn phase. Interestingly, Arabidopsis genes associated with protein synthesis are preferentially expressed at around dawn when plants are exposed to thermocycles, but at midday in photocycles [26]. Since the maize plants used here did not experience thermocycles, maize plants may organize proteins synthesis at a time of day distinct from Arabidopsis. These findings show the maize circadian clock coordinates the expression of major cellular processes to occur at defined times within a normal 24-hour day. The specific physiological processes where the circadian clock plays a large role in coordinating transcription are explored in more depth below.

### Coordinated circadian expression of key C4 photosynthesis genes

**T**he maize proteins encoded by the transcripts represented on the array were given a functional annotation by matching amino acid sequences to likely orthologs in rice and Arabidopsis (Additional file 6 Table S3). The functional annotation grouped genes into a high confidence set and a low confidence set based on the criteria described in "Methods" and outlined in Additional file 4 Figure S3. Importantly, the high confidence genes represent those genes where orthologs were identified in both Arabidopsis and rice. The genes in this high confidence set were used to explore the influence of the circadian clock on maize physiology, in particular maize metabolic and signaling pathways.

**Figure 3:**
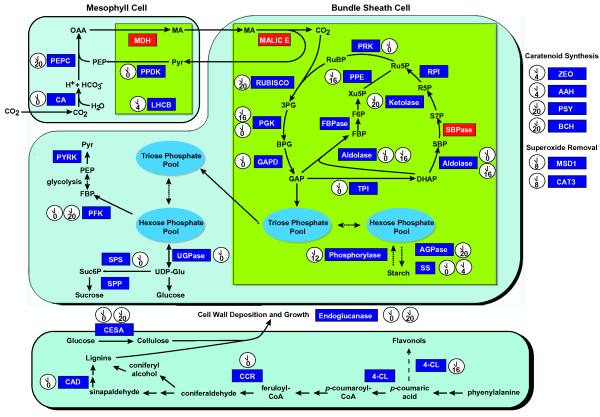
**The circadian clock organized expression of maize photosynthesis, carbohydrate metabolism, and cell wall synthesis genes**. Maize genes encoding enzymes associated with the CO _2 _capture steps of C4 photosynthesis, which take place in mesophyll cells, showed coordinated expression phased to morning (ZT20 and ZT0) and midday (ZT4). Expression of genes encoding enzymes of the Calvin cycle, which occurs in bundle sheath cells, was generally phased to early morning (ZT16 and ZT20). Expression of genes encoding enzymes involved in cell wall synthesis, which are illustrated in a separate cell for clarity, occurred in the morning (ZT20 and ZT0). Large light blue shapes represent cells and green boxes within cells represent chloroplasts. Small dark blue boxes indicate genes on the array and red boxes are genes absent from the array. White clock faces next to enzymes indicate the circadian expression of the gene encoding that enzyme and the number within the clock face shows the expression phase in ZT.

The contribution of the maize circadian clock to photosynthesis was of special interest for its potential to provide insight into circadian regulation of C4 photosynthesis. Many key enzymes involved in C4 photosynthesis were under circadian clock control and expression of these were generally phased around dawn (Table 2; Figure 3), similar to Arabidopsis C3 photosynthesis [28]. A gene encoding PEPC, the C4 enzyme that initially captures carbon for photosynthesis [40] (Figure 3), showed early morning phasing (Table 2). This PEPC is a strong candidate for a genuine C4 enzyme, as its amino acid sequence is highly homologous to a PEPC from sorghum and the genome regions around the maize and sorghum genes are syntenic (data not shown). Circadian regulation of a maize *pepc *gene is a novel observation. However, the true impact of the circadian oscillator on PEPC protein abundance remains to be determined, as the rhythms in transcription may not be translated into changes in protein levels. Interestingly, the Arabidopsis transcript encoding a homologous PEPC has a nearly identical phase of expression as the maize *pepc *transcript (Table 2). A maize *ppdk *gene, which encodes the enzyme that furnishes PEPC substrates [41] (Figure 3), and a *ca *gene both reach peak expression at dawn (Table 2). Expression of an Arabidopsis homolog of PPDK also has a phase matching the maize *ppdk *gene. Therefore, the circadian regulatory networks in C4 and C3 plants similarly organize expression of photosynthesis components. Logically, the expression of *pepc*, *ppdk*, and *ca *immediately precedes stomatal opening and the onset of light that stimulates C4 metabolism [42]. Other components of photosynthesis displayed similar anticipatory expression, including transcripts encoding RBCS3B, RCA, and several LHCB proteins, as well as GAPD and CS, which contribute to chlorophyll synthesis (Table 2). Similarly, a large proportion of the enzymes of the Calvin cycle on the array were circadian clock-regulated such that peak expression preceded or occurred at dawn (Figure 3, Table 2). Consistent with the apparently conserved nature of the circadian network structure, the phase of expression for this set of photosynthesis genes was essentially invariant between maize and Arabidopsis (Table 2). Thus, the maize system has incorporated the initial CO_2 _capture of C4 photosynthesis into the overall coordination of photosynthesis that is shared with C3 photosynthesis.

**Table 2:** The maize circadian clock orchestrated coordinated expression of genes involved in multiple key physiological processes

Biological Process					
Maize protein ID	*At *gene ID	*Zm *phase	^**1**^***At *phase**	PlantCyc Pathway	^**2**^**Function *Zm *to *At***
**Photosynthesis**					

GRMZM2G097457	AT4G15530	ZT0	ZT22	C4 carbon dioxide fixation	PPDK
GRMZM2G121878	AT5G14740	ZT0	ZT18	C4 carbon dioxide fixation	CA2
GRMZM2G083841	AT2G42600	ZT20	ZT19	C4 carbon dioxide fixation	PEPC
GRMZM2G105644	AT1G74470	ZT0	ZT3	Chlorophyll a/b Biosynthesis	GGR
GRMZM2G162672	AT3G51820	ZT16	ZT19	Chlorophyll a/b Biosynthesis	CS
GRMZM2G351977	AT2G34420	ZT4	ZT5	Photosystem II	LHCB
GRMZM2G033885	AT3G08940	ZT4	ZT4	Photosystem II	LHCB
GRMZM2G155216	AT2G34420	ZT4	ZT5	Photosystem II	LHCB
GRMZM2G113033	AT5G38410	ZT20	ZT19	Calvin Cycle	RBCS-3B
GRMZM2G162200	AT2G39730	ZT0	ZT1	Calvin Cycle	RCA
GRMZM2G057823	AT2G36460	ZT0	nc	Calvin Cycle	FBAase2
GRMZM2G155253	AT4G38970	ZT20	ZT21	Calvin Cycle	FBAase1
GRMZM2G026807	AT5G61410	ZT0	ZT19	Calvin Cycle	RPE
GRMZM2G431708	AT1G18270	ZT16	ZT7	Calvin Cycle	KPBA
GRMZM2G463280	AT1G32060	ZT16	ZT20	Calvin Cycle	PRK
GRMZM2G033208	AT3G60750	ZT20	ZT16	Calvin Cycle	KETOLASE
GRMZM2G382914	AT1G79550	ZT20	ZT23	Calvin Cycle	PGK
GRMZM2G065928	AT3G19270	ZT4	nc	Carotenoid Biosynthesis	AAH
GRMZM2G127139	AT5G67030	ZT4	ZT2	Carotenoid Biosynthesis	ZEO
GRMZM2G454425	AT4G25700	ZT20	ZT21	Carotenoid Biosynthesis	BCH1
GRMZM2G149317	AT5G17230	ZT20	ZT22	Carotenoid Biosynthesis	PSY
GRMZM2G124455	AT3G10920	ZT8	nc	Superoxide Radical Removal	MSD1
GRMZM2G079348	AT1G20620	ZT8	ZT11	Superoxide Radical Removal	CAT3

**Carbohydrate Metabolism**					

GRMZM2G080375	AT4G26270	ZT0	ZT15	Glycolysis	PFK3
GRMZM2G132069	AT4G26270	ZT20	ZT15	Glycolysis	PFK3
GRMZM2G127717	AT2G22480	ZT20	ZT5	Glycolysis	PFK5
GRMZM2G051004	AT1G16300	ZT0	ZT23	Glycolysis	GADP-2
GRMZM2G089136	AT1G56190	ZT20	ZT19	Glycolysis	PGK
GRMZM2G002807	AT2G21170	ZT16	nc	Glycolysis	TPI
GRMZM2G079888	AT5G66760	ZT0	nc	TCA cycle	SDH1
GRMZM2G055331	AT5G20280	ZT0	nc	Sucrose Biosynthesis	SPS1F
GRMZM2G032003	AT5G17310	ZT0	ZT0	Sucrose Degradation	UDPase
GRMZM2G089836	AT1G62660	ZT0	nc	Sucrose Degradation	BFRUCT3
GRMZM2G084694	AT1G56560	ZT20	nc	Sucrose Degradation	BFRUCT
GRMZM2G391936	AT5G19220	ZT20	nc	Starch Biosynthesis	AGPL1
GRMZM2G126988	AT3G01180	ZT0	ZT2	Starch Biosynthesis	SS2
GRMZM2G008263	AT1G32900	ZT4	ZT1	Starch Biosynthesis	SS
GRMZM2G147770	AT3G46970	ZT12	ZT12	Starch Degradation	PHS2
AC207628.4_FGP006	AT1G69830	ZT16	ZT11	Starch Degradation	AMY3
					
**Cell Wall**					

GRMZM2G055795	AT4G18780	ZT20	nc	Cellulose Biosynthesis	IRX1
GRMZM2G142898	AT5G17420	ZT0	nc	Cellulose Biosynthesis	IXR3
GRMZM2G445905	AT5G44030	ZT20	nc	Cellulose Biosynthesis	CESA4
GRMZM2G113137	AT5G64740	ZT20	ZT21	Cellulose Biosynthesis	CESA6
GRMZM2G052149	AT3G03050	ZT20	nc	Cellulose Biosynthesis	CSLD3
GRMZM2G477603	AT1G65610	ZT20	ZT0	Cellulose Metabolism	GUN7
GRMZM2G110735	AT5G49720	ZT0	nc	Cellulose Metabolism	GUN25
GRMZM2G131205	AT1G15950	ZT0	ZT1	Lignin Biosynthesis	CCR1/IXR4
GRMZM2G110175	AT3G19450	ZT0	ZT22	Lignin Biosynthesis	CAD4
GRMZM2G054013	AT1G65060	ZT16	ZT21	Flavonol Biosynthesis	4-CL3

**Growth and Development**					

GRMZM2G164405	AT4G11280	ZT0	nc	Ethylene Biosynthesis	ACS6
GRMZM2G117198	AT3G17390	ZT20	nc	Ethylene Biosynthesis	MTO3
GRMZM2G081554	AT4G02780	ZT4	nc	Gibberellin Biosynthesis	GA1
GRMZM2G093195	AT2G32440	ZT16	ZT8	Gibberellin Biosynthesis	KAO2
GRMZM2G147882	AT1G35190	ZT20	ZT2	Gibberellin Biosynthesis	OR
GRMZM2G001457	AT3G45780	ZT8	ZT7	Blue light response	PHOT1
GRMZM2G067702	AT3G46640	ZT12	ZT14	Circadian Clock	LUX
GRMZM2G095727	AT5G02810	ZT8	ZT7	Circadian Clock	PRR7
GRMZM2G107101	AT1G22770	ZT12	ZT11	Circadian Clock/Flowering	GI1A
GRMZM2G149786	AT3G13682	ZT12	nc	Flowering	LDL1
GRMZM2G065276	AT4G16280	ZT12	nc	Flowering	FCA
GRMZM2G405368	AT5G15850	ZT16	ZT1	Flowering	COL1

^1^Phase of Arabidopsis transcript expression from the DIURNAL web tool [71], where phase is determined with HAYSTACK [35]. "nc" indicates noncycling transcript with a waveform assigned a PCC < 0.7 by HAYSTACK analysis.

^2^Function of maize protein inferred from the function of the Arabidopsis ortholog according to Kyoto Encyclopedia of Genes and Genomes [70].

The state of the maize circadian system observed here is likely an incomplete representation of the network in field grown mature plants, since young maize seedlings are developing their C4 photosynthetic capacity. Burris and de Veau showed that while 9 day-old maize seedlings exhibited a 3 times higher rate of photorespiration than 3 month-old maize plants, consistent with limited C4 photosynthesis in seedlings compared to the mature plants, the rate of photorespiration in these maize seedlings was 5 to 7 times lower than mature wheat plants performing C3 photosynthesis [43]. Their analysis demonstrates young maize seedlings have the capacity for C4 photosynthesis, albeit a modified version with a higher rate of photorespiration than mature plants. Therefore, 7 day-old maize seedlings represent a suitable model of C4 photosynthesis.

Circadian expression was evident for genes encoding enzymes involved in biosynthesis of carotenoids including PSY, BCH, and ZEO (also known as ABA DEFICIENT 1 in Arabidopsis) (Table 2, Figure 3). Carotenoids serve as photoprotective pigments and are also important structural components of light harvesting complexes. PSY catalyzes the first and rate-limiting step in the production of carotenoids and both BCH and ZEO lie downstream of PSY [44,45]. Expression of these genes was phased to either early morning (ZT20) or midday (ZT4), clearly showing the maize circadian system orchestrates expression of upstream and downstream enzymes for carotenoid production to coincide with the time when these compounds are needed for photosynthesis and non-photochemical quenching. PSY is also circadian regulated in Arabidopsis [25], with a phase matching that found here for maize (Table 2), which again suggests strong conservation of the regulatory networks underlying the maize transcriptome. Protection from ROS produced by photosynthesis is provided by the action of catalase and superoxide dismutase enzymes. Genes encoding maize CAT3 and an ortholog of MSD1 exhibited circadian expression in maize seedlings (Table 2). Consistent with their role in scavenging ROS arising from photosynthesis, expression of the *cat3 *and *msd1 *genes reached peak levels during late day (ZT8) when ROS is more likely to accumulate (Figure 3). Overall, these findings show the circadian clock organizes maize C4 photosynthesis to ensure the plant makes maximal use of light energy available during the day.

### Organization of carbohydrate metabolism gene expression by the maize circadian clock

The maize clock also regulates genes contributing to carbohydrate metabolism and carbon flux (Figure 3, Table 1). For example, the circadian clock controlled expression of two PFK encoding genes and peak expression for each occurred around dawn (Figure 3). Note that the annotation matched two different maize transcripts to the same Arabidopsis gene (PFK3), which explains the different phase values shown in Table 2. PFK enzymes mediate the central regulatory step of glycolysis. Two glycolysis enzymes of the FBAase class, which reversibly convert fructose bisphosphate to triose phosphate, were clock regulated such that one peaked in the morning (FBAase2) and another expressed in the evening (FBAase1). As in energy generation, the clock regulates genes involved in energy storage. AGPase is the major regulatory enzyme in starch biosynthesis, where it converts glucose 6-phosphate to ADP-glucose, the substrate for starch synthase [46]. The transcript encoding AGPL, a subunit of AGPase, reached peak levels near dawn at ZT20 (Figure 3). Early morning accumulation of AGPase may be an anticipatory strategy to prepare the system for midday when photosynthate is in excess and starch biosynthesis commences. Similarly, two *ss *transcripts encoding starch synthases were maximally expressed early in the day (ZT0 and ZT4) like their Arabidopsis orthologs (Table 2). Thus, the maize circadian clock anticipated the need for carbon metabolism components and up-regulated expression of these genes so that enzymes would be present at the time when photosynthate would be available for energy production and storage. Together, the coordinated regulation of carbohydrate metabolism and C4 photosynthesis indicates that the circadian clock in maize organizes gene expression to ensure efficient and maximal energy production, use, and storage throughout the day.

### Co-regulation of cell wall synthesis genes by the maize circadian clock

Cell walls are a major constituent of plant biomass, and their enlargement exhibits a biological rhythm [47,48]. Consistent with rhythmic growth, the transcripts of several enzymes involved in cell wall biosynthesis were found to be circadian-regulated in maize (Table 2, Figure 3). CCR and CAD catalyze two of the final steps in the conversion of phenylpropanoid into monolignins to achieve wall hardening [49]. Transcripts encoding both these enzymes were found to have cyclic expression peaking at dawn (Figure 3). The maize CAD is orthologous to Arabidopsis CAD4. Arabidopsis CAD4 is a class II CAD enzyme that acts on sinapaldehyde instead of coniferaldehyde [50]; therefore, the substrate for the maize CAD is likely to be sinapaldehyde (Figure 3). 4-CL acts early in phenylpropanoid synthesis and a maize gene encoding an ortholog of Arabidopsis 4-CL3 is rhythmically expressed with peak expression at ZT16, instead of ZT0 like the *ccr *and *cad *transcripts. Since the Arabidopsis 4-CL3 enzyme participates in flavonoid biosynthesis instead of contributing to lignin production [50], this maize 4-CL is likely not involved in lignin biosynthesis but flavonoid biosynthesis instead. The similar expression waveforms of *ccr *and *cad *in maize and Arabidopsis suggests the timing of this aspect of lignin biosynthesis is conserved (Table 2) [28]. Several cellulose synthase genes shared nearly the same dawn phasing as the *ccr *and *cad *transcripts (Figure 3). Similarly, transcripts for two endoglucanases and a cellulase cycled at dawn in the maize dataset (Figure 3). Circadian dawn expression of cell wall-related enzymes correlates with the time of maximal plant growth rate reported for Arabidopsis [49,51]; therefore, the maize circadian clock seems to regulate daily cell wall construction so that it coincides with growth trigged by phytohormone signaling.

### Circadian clock regulation of maize GA, ethylene, and ABA biosynthesis genes

Several recent studies have shown a fundamental role of the Arabidopsis circadian clock is to indirectly control growth and development through transcriptional regulation of genes involved in phytohormone biosynthesis and response [25,27,29]. Placement of the maize cycling genes in the context of metabolic pathways revealed that the maize circadian clock also regulates genes involved in the synthesis of phytohormones fundamental to plant growth (Table 2).

**Figure 4:**
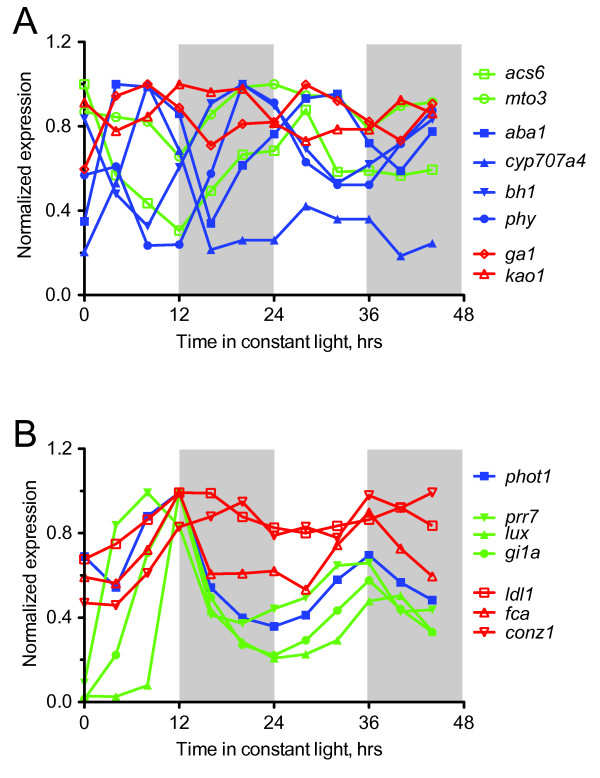
**Genes associated with phytohormone biosynthesis, flowering time, phototropism, and the circadian clock exhibited rhythmic expression**. A) Expression waveforms of high confidence cycling biosynthesis genes for ethylene (green open symbols), ABA (blue closed symbols) and GA (red open symbols). B) Expression waveforms of high confidence cycling genes for phototropism (blue closed squares), circadian clock (green closed symbols), and flowering time (red open symbols). Shaded regions represent subjective night. For each gene, the normalized expression values from the array were further normalized to the highest expression value for that gene.

GAs and ethylene are important positive regulators of plant cell growth. GAs promote longitudinal expansion of cells and ethylene promotes transverse cell expansion [52]. Daily rhythmic growth exhibited by Arabidopsis is, in part, controlled through diurnal transcriptional control of genes encoding enzymes for synthesis of GAs and ethylene [29]. For GAs, GA _12 _biosynthesis is a prerequisite for the production of active GAs. In Arabidopsis, GA1, which encodes *ENT*-COPALYL DIPHOSPHATE SYNTHASE, and KAO2 act in the biosynthetic pathways that provide the GA _12 _precursor of GA synthesis [53,54]. Maize orthologs of GA1 and KAO2 were among the proteins encoded by circadian expressed transcripts (Table 2). Maize *ga1 *reached peak expression at subjective midday (Figure 4A), consistent with the phasing reported for GA synthesis genes in Arabidopsis seedlings under constant light conditions [25]; however, Arabidopsis *GA1 *does not exhibit circadian expression (Table 2). Maize *kao2 *showed peak circadian expression at ZT16, which was 12 hours later than maize *ga1 *and 8 hours later than its Arabidopsis counterpart (Figure 4A). Nevertheless, the robust circadian expression of *ga1 *and *kao1 *indicates the participation of the maize circadian clock in the coordination of maize growth through regulation of phytohormone biosynthesis. In support of this possibility, two putative ethylene biosynthesis genes also showed circadian expression: orthologs of Arabidopsis MTO3 and ACS6. MTO and ACS enzymes act sequentially to produce ACC, which is the precursor to ethylene [55]. The dawn-phased expression of the maize *acs6 *and *mto3 *genes was in contrast to the non-circadian expression for their Arabidopsis orthologs (Table 2). The lack of correspondence in the phasing of GA and ethylene synthesis between maize and Arabidopsis may be a consequence of the constant light condition used here. Along with the circadian clock, diurnal light and dark cues serve an important role in determining the phase of phytohormone synthesis genes and ultimately the timing of cellular growth [26,51]. Therefore, evaluation of maize expression in diurnal conditions will be important to fully comprehend the molecular basis for rhythmic growth in maize.

The plant stress hormone ABA antagonizes the growth promoting effects of GA and ethylene [52] and, like GAs and ethylene, ABA biosynthesis genes are part of a transcriptional regulatory network that exerts daily control over plant growth [26]. The carotenoid biosynthesis pathway supplies precursors for the biosynthesis of ABA [56]. As noted above, the transcript encoding PSY, the enzyme governing the first committed step in carotenoid synthesis, was under circadian control with peak expression in the early morning (Table 2, Figure 4A). Several genes downstream of PSY that are involved in ABA synthesis also showed dawn expression (Table 2, Figure 4A), which is consistent with ABA synthesis taking place in the early morning. Comparable circadian regulation of PSY and genes encoding downstream components of this pathway has been noted in Arabidopsis [25]. The conserved circadian regulation of maize carotenoid biosynthesis genes observed here suggests that synthesis of ABA in maize leaves is under circadian control, which likely impacts the key role ABA plays in antagonizing cell growth and in stimulating stomatal closure [57]. The latter role of ABA is associated higher-water use efficiencies in crop plants, since closure of stomata reduces water loss. Therefore, the maize circadian system is likely a critically important, but under appreciated, contributor to maize productivity in the field.

### Rhythmic expression of putative maize flowering time, circadian clock, and phototropism genes

Several orthologs of Arabidopsis circadian clock and flowering time genes were present in the maize cycling gene set; however, the array had limited overall representation of genes in these categories. Included in the rhythmic collection was the gene encoding CONZ1, which the annotation matched to Arabidopsis COL1 (Table 2, Figure 4B). CONZ1 is a strong candidate for the functional ortholog of Arabidopsis CONSTANS and rice Heading date 1 [36]. CONSTANS and Heading date 1 serve in control of flowering time to regulate the expression of the FT class of proteins. FT proteins act as florigen molecules that promote floral development at the shoot apical meristem [6]. *gi1A *and *gi1B *are paralogous flowering time and circadian clock genes identified in the same study as *conz1 *[36]. A probe set for *gi1A *was on the array and this transcript exhibited a waveform and phase similar to that of Arabidopsis *GI *expression (Figure 4B, Table 2) [58]. Orthologs of Arabidopsis LDL1 and FCA were two other potential flowering time transcripts with rhythmic expression in the maize dataset (Figure 4B). LDL1 and FCA are known to act in the Arabidopsis autonomous flowering pathway [59]. LDL1 is involved in modification of chromatin and, therefore, is not likely to be strictly involved in flowering time [60]. Similarly, FCA plays a role in RNA-mediated silencing through DNA methylation [61]. Circadian clock components present on the array included orthologs of Arabidopsis PRR7 and LUX (Table 2, Figure 4B). Expression of maize *prr7 *and *lux *exhibited the same phasing as their Arabidopsis orthologs (Table 2), indicating the conserved nature of the core maize circadian clock. Finally, the maize *phot1 *transcript, encoding a blue light photoreceptor of the phototropin family likely to be involved in phototropism, was robustly rhythmic with the identical phase as its Arabidopsis counterpart (Figure 4B). This restricted sampling of flowering time, circadian clock, and phototropism genes suggests that maize relies on conserved signaling networks for these key processes.

## Conclusions

Identification of maize genes under circadian clock regulation and the predicted contribution of these genes to metabolism, growth, and development indicates the maize circadian clock plays an important role in coordinating the overall physiology of this C4 crop plant. In general, circadian regulation of the enzymes mediating C4 photosynthesis was predictable based on the Arabidopsis C3 model and without any apparent large-scale changes to accommodate the specialized C4 anatomy and enzymology. Recent investigation of the global effects of the circadian system on Arabidopsis physiology demonstrated that rhythms are critical to plant fitness and optimal growth [8]. A more complete appreciation of the maize circadian clock will reveal where the circadian system impacts maize growth and development, as well as highlight novel approaches for optimization of crop production through targeted modification of the circadian system.

## Methods

### Plant materials and growth conditions

Seeds of inbred B73 were obtained from the Maize Genetics Cooperation Center. B73 is widely used and is the source material for the complete maize genome sequence [31]. Seedlings were germinated and grown in 57 × 30 × 6 cm flats filled with Scotts Supersoil (scotts.com) inside a Conviron (conviron.com) growth chamber at constant 26°C and constant 70% humidity. High intensity cool white fluorescent bulbs provided illumination at a fluence rate of 200 μmoles m^-2 ^s^-1^. Seedlings were grown for seven days under 12 hours of light followed by 12 hours of dark before being transferred to LL for two days at the same temperature and humidity.

### RNA sample preparation and array hybridization

Tissue harvest began following transfer of seedlings to LL on the 8^th ^day. The entire aerial portion (corresponding to all tissue above the prop roots) of five seedlings was harvested beginning at dawn and every 4 hours thereafter for the next 48 hours. Samples were immediately frozen in liquid nitrogen. Three separate experimental replicates were collected in this way. Tissue was ground to a fine powder under liquid nitrogen and total RNA isolated by Trizol extraction (Invitrogen; invitrogen.com) followed by Qiagen RNeasy columns and treatment with RNase-free DNase I (Qiagen; qiagen.com). cRNA was generated from total RNA of three pooled replicate samples with the GeneChip^® ^One-Cycle Target Labeling kit according to the manufacturer's recommendations (Affymetrix, affymetrix.com). The University of California, Berkeley Functional Genomics Laboratory [62] hybridized samples to Affymetrix GeneChip^® ^Maize Genome Arrays and scanned the washed arrays as suggested by manufacturer. Present, Marginal, and Absent calls for each probe set were determined with MAS5 analysis [63] and these are presented in Additional file 7 Table S4. Probe sets called "Absent" at more than nine time points were removed from the downstream analysis [26]. Raw hybridization intensities were normalized across all twelve arrays using RMAExpress in Perfect Match mode [64,65]. The raw microarray data is available online as experiment ZM28 at the Plant Expression Database [66].

### Identification of maize transcripts with circadian expression waveforms

Rhythmic expression waveforms within the normalized dataset were identified by analysis with the COSOPT curve fitting algorithm and the pattern matching function HAYSTACK. The COSOPT algorithm is a cosine waveform fitting method that identifies transcripts with circadian expression by first performing an arithmetic linear-regression detrend of the normalized time series data for each transcript followed by testing detrended data for a fit to 101 cosine test models [32]. An approximate goodness of fit value produced by COSOPT analysis is MMC-β, where lower values correspond to a better fit to an ideal cosine curve. Based on previous studies in Arabidopsis [25,26,28,33] and our empirical tests, a MMC-β threshold of ≤ 0.05 was chosen to call a given probe set circadian. HAYSTACK is a model-based, pattern-matching tool that identifies rhythmic expression patterns by aligning time-series microarray data to a set of discrete diurnal and circadian models [35]. HAYSTACK conducts a least square linear regression for each gene against all the models and calculates the best-fit model, phase-of-expression, and p-value for each gene. HAYSTACK was run on the normalized maize dataset with the following parameters: fold cutoff = 2, p-value cutoff = 0.05, background cutoff = 50, and PCC = 0.7 with the 552 model set described in Michael *et al*. 2008 [26]. In general, goodness of fit between the model and experimental traces is revealed by the PCC value. We empirically determined that a PCC ≥ 0.7 produced good matches between model and experimental data. This cutoff was less stringent than that applied in previous studies investigating Arabidopsis circadian expression [26,29].

### Phase clustering of circadian waveforms

Probe sets with similar expression waveforms were grouped together by K-means clustering. The RMAExpress normalized values for probe sets with MMC-β ≤ 0.05 and those with PCC ≥ 0.7 were converted to normalized values with the following calculation: value = [(expression at single time point) - mean(all time points)]/[standard deviation(all time points)]. These values were used to fit the waveforms to six clusters representing each ZT phase with the K-means clustering algorithm built into the TIGR Multiexpression Viewer tool [67]. Clustering ran for a total of 50 iterations and was considered complete when probe sets were no longer exchanged between clusters. In two independent trials, no greater than 7 iterations were required to build the clusters.

### Matching of probe sets to maize genes, assignment of GO Slim terms to genes, and functional annotation

Additional file 4 Figure S3 outlines the annotation scheme used to match probe sets to genes in the maize genome, identify GO Slim terms for maize genes, and functionally annotate maize genes. Transcripts represented on the Affymetrix GeneChip^® ^Maize array were mapped to maize transcripts with a custom Perl script that used the DNA sequences provided by Affymetrix and BLASTn to query the ZmB73 4.53a filtered CDS sequences [31]. After the maize gene ID was identified for each probe set, GO Slim terms and associated PlantCyc pathways [68] were extracted from the Maize Genome Consortium website [69]. Maize gene IDs for each gene were used with a local implementation of BLASTp to identify likely rice orthologs in the Michigan State University Rice Pseudomolecule and Genome Annotation release 6.1 and likely Arabidopsis orthologs in TAIR9_pep_20090619 sequences. The output of these queries were separated into high and low confidence lists (Additional file 6 Table S3). Criteria for the high confidence list were amino acid identity of 40% or greater, Highest Scoring Segment Pair (HSP) of 50 amino acids or greater, and a match to putative orthologs in both Arabidopsis and rice. The low confidence list contains the remaining genes that encode proteins not meeting these three criteria. Putative functions of matched orthologs were derived from Kyoto Encyclopedia of Genes and Genomes [70]. Phase of expression for Arabidopsis orthologs shown in Table 2 was determined with the DIURNAL tool from the "LL12" dataset and matches between model and data considered significant at PCC values ≥ 0.7 [71]. Overrepresentation of GO Slim terms was calculated with hypergeometric distribution followed by a modified Bonfferoni correction for multiple testing as implemented in GeneMerge [72]. GeneMerge produced an e-score comparable to a P-value that was used to determine significance of overrepresentation. GO Slim terms for the entire array were the population set and the indicated collection of cycling genes served as the study set. Overrepresented GO Slim terms were considered those with e-scores < 0.05.

## List of Abbreviations

4-CL: 4-COURMARATE-COA LIGASE; 3PG: 3-phosphoglycerate; AAH: ABSCISIC ACID 8-HYDROXYLASE; ABA: abscisic acid; ACC: 1-AMINOCYCLOPROPANE-1-CARBOXYLIC ACID; ACS: ACC SYNTHASE; AGPase: ADP-GLUCOSE PYROPHOSPHORYLASE; AGPL: ADP-GLUCOSE PYROPHOSPHORYLASE LARGE SUBUNIT; AMY: ALPHA-AMYLASE-LIKE; BCH: BETA-CAROTENE HYDROXYLASE; BPG: 1:3-bisphosphoglycrate; CA: CARBONIC ANHYDRASE; CAD: CINNAMYL ALCOHOL DEHYDROGENASE; CAT3: CATALASE 3; CCA1: CIRCADIAN CLOCK ASSOCIATED 1; CESA: CELLULOSE SYNTHASE; CHE: CCA1 HIKING EXPEDITION; CS: CHLOROPHYLL SYNTHETASE; CSLD: CELLULOSE SYNTHASE-LIKE; CCR: CINNAMOYL-COA REDUCTASE; COL: CONSTANT-LIKE; CONZ1: CONSTANS OF ZEA MAYS1; DHAP: dihydroxyacetone phosphate; ELF3: EARLY FLOWERING 3; GA: Gibberellin; GA1: GA REQUIRING 1; GAP: glyceraldehyde 3-phosphate; GAPD: GLYCERALDEHYDE-3-PHOSPHATE DEHYDROGENASE; GGR: GERANYLGERANYL REDUCTASE; GI: GIGANTEA; GO:  Gene Ontology; F6P: fructose-6-phosphate; FBA: 1:6-BISPHOSPHATE ALDOLASE; FBP: fructose-1;6-bisphosphate; FBPase: FRUCTOSE BISPHOSPHATASE; FRUCT: BETA-FRUCTOSIDASE; FT: FLOWERING LOCUS-T; GUN: ENDO-1:4-β-GLUCANASE; IXR: IRREGULAR XYLEM; KAO2: ENT-KAURENOIC ACID HYDROXYLASE 2; KBPA: KETOSE-BISPHOSPHATE ALDOLASE CLASS II; KETOLASE: TRANSKETOLASE; LDL1: LYSINE-SPECIFIC DEMETHYLASE 1; LHCB: LIGHT HARVESTING COMPLEX B; LHY: LATE ELONGATED HYPOCOTYL; LL: constant light; LUX: LUX ARRHYTHMO; MA: malate; MDH: MALATE DEHYDROGENASE; MALIC E: MALIC ENZYME; MMC-β: Multiple Measures Corrected-β; MSD1: MANGANESE SUPEROXIDE DISMUTASE1; MTO3: METHIONINE OVER-ACCUMULATOR 3; OAA: oxaloacetate; OR: OXIDOREDUCTASE; PCC: Pearson Correlation Coefficient; PEP: phospho *enol *pyruvate; PEPC: PHOSPHO *ENOL *PYRUVATE CARBOXYLASE; PFK: 6-PHOSPHOFRUCTOKINASE; PGK: PHOSPHOGLYCERATE KINASE; PHOT: PHOTOTROPIN; PHS2: ALPHA-GLUCAN PHOSPHORYLASE; PPDK: PYRUVATE; ORTHOPHOSPHATE DIKINASE; PPE: PHOSPHOPENTOSE EPIMERASE; PRK: PHOSPHORIBULOKINASE; PRR: PSEUDO-RESPONSE REGULATOR; PSY: PHYTOENE SYNTHASE; Pyr: pyruvate; PYRK: PYRUVATE KINASE; R5P: ribose-5-phosphate; RBCS: RUBISCO SMALL SUBUNIT; RCA: RUBISCO ACTIVASE; ROS: reactive oxygen species; RPE: RIBULOSE-PHOSPHATE 3-EPIMERASE; RPI: RIBOSE PHOSPHATE ISOMERASE; Ru5P: ribulose-5-phosphate; RUBISCO: RIBULOSE BISPHOSPHATE CARBOXYLASE OXYGENASE; RuBP: ribulose-1:5-bisphosphate; S7P: sedoheptulose-7-phosphate; SBP: sedoheptulose-1:7-bisphosphate; SBPase: SEDOHEPTULOSE BISPHOSPHATASE; SDH: SUCCINATE DEHYDROGENASE; SPP: SUCROSE-PHOSPHATE PHOSPHATASE; SPS: SUCROSE-PHOSPHATE SYNTHASE; SS: STARCH SYNTHASE; Su6P: sucrose 6-phosphate; suc: sucrose; TOC1: TIMING OF CAB EXPRESSION 1; TPI: TRIOSE PHOSPHATE ISOMERASE; UDP-Glu: Uridine diphosphate glucose; UGPase: UDP-GLUCOSE PYROPHOSPHORYLASE; Xu5P: xylulose-5-phosphate; ZEO: ZEAXANTHIN EPOXIDASE; ZT: Zeitgeber Time; ZTL: ZEITLUPE.

## Authors' contributions

SK carried out the microarray analysis, annotation, comparative analysis, and drafted the manuscript. SCR helped draft the manuscript and created figures. FGH conceived the study, participated in its design and coordination, created figures, and drafted the manuscript. All authors read and approved the final manuscript.

## Supplementary Material

Additional file 1 **Normalized expression values for each probe set across the complete 44-hour time course**. MMC-β values from COSOPT analysis and PCC values for HAYSTACK analysis. Click here for file

Additional file 2 **Transcripts expected to be under circadian control were present in the maize cycling transcriptome**. COSOPT and HAYSTACK captured bona-fide rhythmically expressed transcripts including likely maize flowering time genes *conz1 *(purple circles) and *gi1a *(green squares), as well as established circadian clock-regulated genes *cat3 *(blue open triangles) and three *lhcb *transcripts (red closed symbols). Shaded squares represent subjective night. For each gene, the normalized expression values = [(expression at single time point) - mean(all time points)]/[standard deviation(all time points)]. Click here for file

Additional file 3 **Phase clusters of genes with similar circadian expression waveforms**. Transcript expression profiles were placed into one of six phase bins by K-means clustering (see "Methods"). The number of transcripts in each phase cluster is indicated next to the time of peak expression in ZT for that group. Gray regions represent subjective night. Click here for file

Additional file 4 **Functional annotation method used to match probe sets on the Affymetrix GeneChip^® ^Maize Genome Array to maize genes and to identify orthologs in Arabidopsis and rice**. Click here for file

Additional file 5 **Probe sets matched to maize gene IDs in the v.ZmB73 4.53a filtered CDS sequence set from the Maize Genome Sequencing Consortium and annotation of these gene IDs with GO Slim terms and PlantCyc pathways**. Click here for file

Additional file 6 **Functional annotation of maize transcripts represented on the microarray by identification of orthologous proteins from *Oryza sativa *and *Arabidopsis thaliana***. High and low confidence genes were determined as described in "Methods". The high and low confidence gene sets are segregated into separate worksheets. Click here for file

Additional file 7 **Present/Marginal/Absent (PMA) calls for probe sets on each of the twelve microarrays**. Click here for file
