# Supplementary figures and images for: Coordination of the maize transcriptome by a conserved circadian clock
Source: BMC Plant Biol. 2010 Jun 24;10:126. doi: 10.1186/1471-2229-10-126 (PMC3095283; doi:10.1186/1471-2229-10-126)

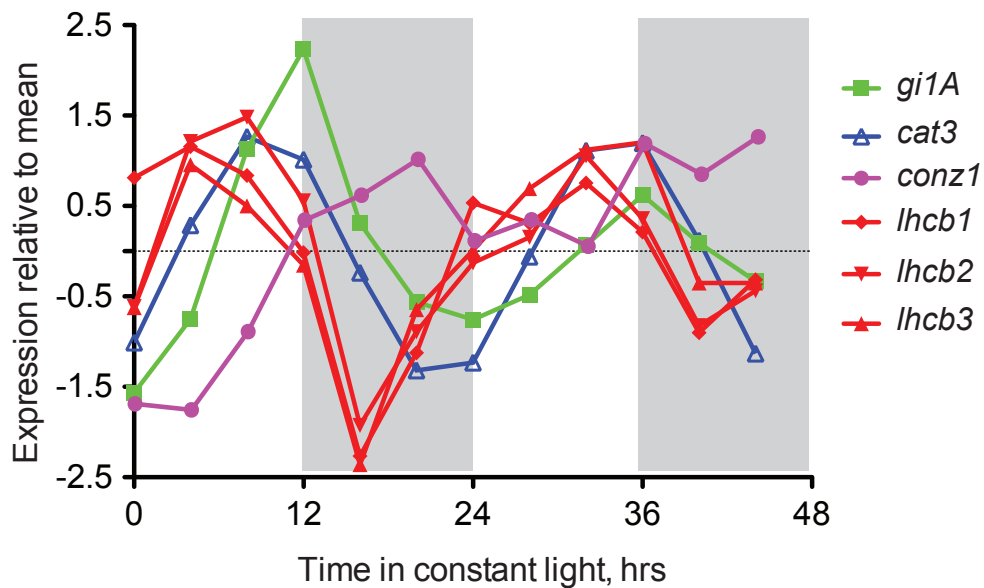

Supplement: Additional file 2 — Transcripts expected to be under circadian control were present in the maize cycling transcriptome. COSOPT and HAYSTACK captured bona-fide rhythmically expressed transcripts including likely maize flowering time genes conz1 (purple circles) and gi1a (green squares), as well as established circadian clock-regulated genes cat3 (blue open triangles) and three lhcb transcripts (red closed symbols). Shaded squares represent subjective night. For each gene, the normalized expression values = [(expression at single time point) - mean(all time points)]/[standard deviation(all time points)]. [file 1471-2229-10-126-S2.PDF]

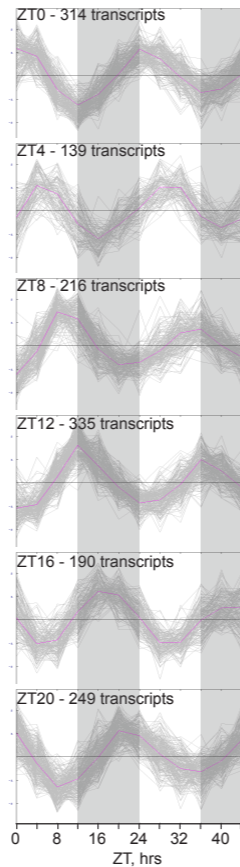

Supplement: Additional file 3 — Phase clusters of genes with similar circadian expression waveforms. Transcript expression profiles were placed into one of six phase bins by K-means clustering (see "Methods"). The number of transcripts in each phase cluster is indicated next to the time of peak expression in ZT for that group. Gray regions represent subjective night. [file 1471-2229-10-126-S3.PDF]

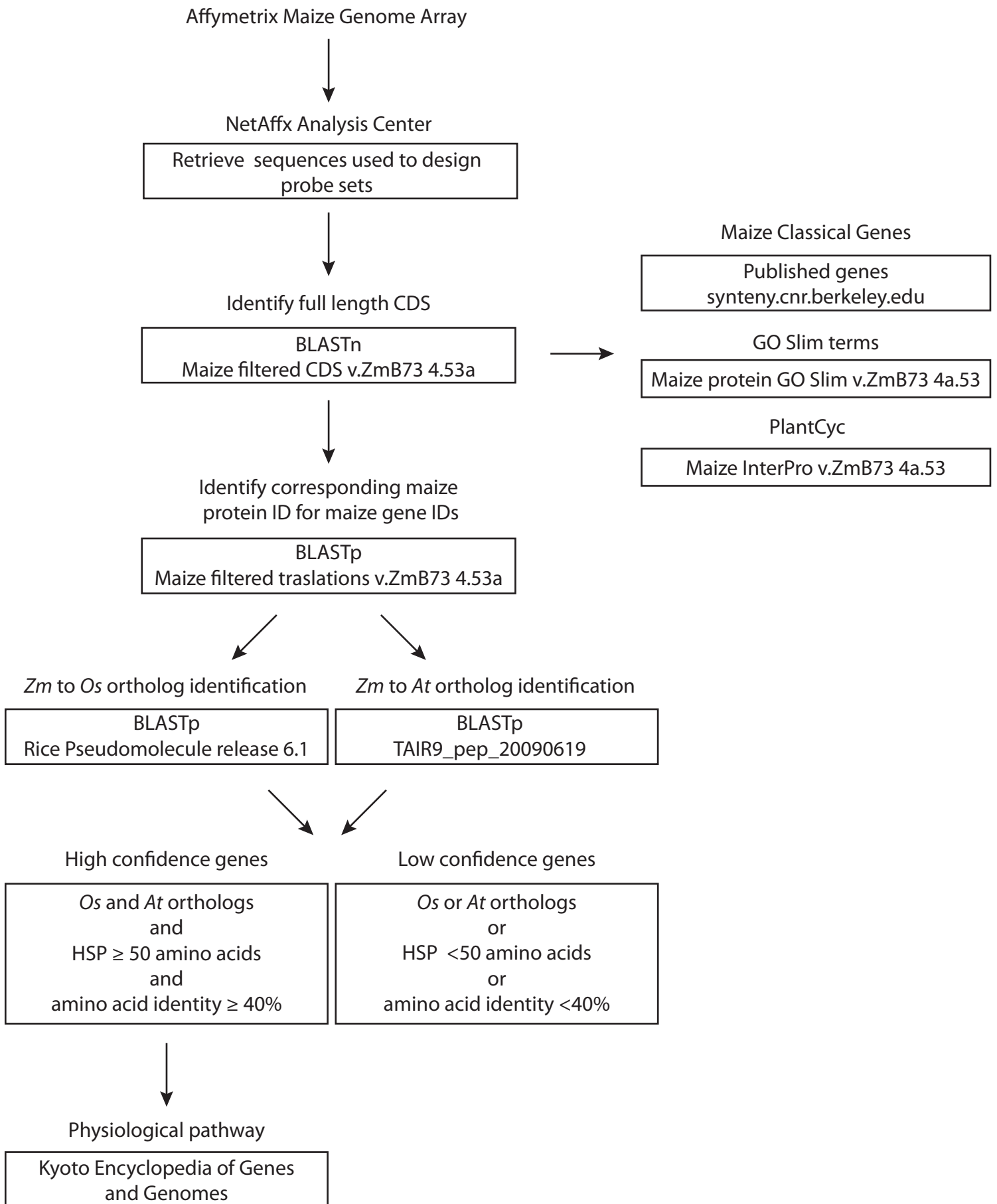

Supplement: Additional file 4 — Functional annotation method used to match probe sets on the Affymetrix GeneChip® Maize Genome Array to maize genes and to identify orthologs in Arabidopsis and rice. [file 1471-2229-10-126-S4.PDF]
